# Acute Rheumatic Fever Presenting as a Mimicker of Septic Arthritis

**DOI:** 10.7759/cureus.9431

**Published:** 2020-07-27

**Authors:** Ikechukwu Achebe, Kifah Hussain, Annette Abraham, Jennifer C Asotibe, Hafeez Shaka

**Affiliations:** 1 Internal Medicine, John H. Stroger, Jr. Hospital of Cook County, Chicago, USA

**Keywords:** septic arthritis, rheumatic heart disease, post strep reactive arthritis, acute rheumatic fever, prophylaxis, migratory arthralgia, arf, psra, rhd

## Abstract

Acute rheumatic fever (ARF) describes the non-suppurative and autoimmune inflammation of joint, muscle, and fibrous tissue that occurs after group A streptococcal (GAS) pharyngitis. This report describes a rare case of a 39-year-old male with migratory arthralgias as a presenting sign of ARF. Through this case, we review the current literature on ARF and highlight clinical and objective findings that differentiate ARF from similar presenting arthralgias, specifically post-streptococcal reactive arthritis (PSRA). With this report, we hope to increase clinical suspicion for ARF in patients with acute joint pain, as differentiating ARF from other arthritides, PSRA specifically, determines management strategy and need for secondary prophylaxis against rheumatic heart disease.

## Introduction

Acute rheumatic fever (ARF) describes a syndrome of non-suppurative and autoimmune inflammation of joint, muscle, and fibrous tissue that occurs as a sequela of untreated pharyngitis with group A Streptococcus pyogenes (GAS) [1]. In this report, we discuss the rare case of a 39-year-old male with migratory arthralgias as a presenting sign of ARF. We also aim to review current literature and highlight diagnostic challenges that occur when differentiating atypical ARF from its mimickers. With this report, we hope to increase clinical suspicion for ARF, as early detection, treatment, and prophylaxis can prevent long-term complications.

## Case presentation

The patient described is a 39-year-old male with no medical history who presented with left wrist pain, swelling, and fever for one day. The pain was unprovoked, abrupt in onset, and constant. On review of systems, the patient denied any history of trauma, urethritis, conjunctivitis, recent alcohol use, and or any tick bites. The patient reported no history of sexually transmitted infections and had not been sexually active for the past year.

A week prior, the patient presented to the emergency department (ED) for similar pain and swelling. During that time, his symptoms localized to his ankles bilaterally. Shortly after receiving ibuprofen in the ED, his symptoms improved and he was discharged on ibuprofen for pain control.

On examination, the patient was febrile with a temperature of 102°F and tachycardic to 129 bpm. His left wrist and metacarpophalangeal (MCP) joints were erythematous, warm to touch, and edematous. The left wrist was tender to palpation, and range of motion was limited by pain and swelling. Examination of the patient’s ankles was unrevealing.

Orthopedics was consulted, and after evaluating the patient, decided against a joint lavage. Arthrocentesis of the left wrist was done, and the patient was empirically started on ceftriaxone and vancomycin.

**Figure 1:**
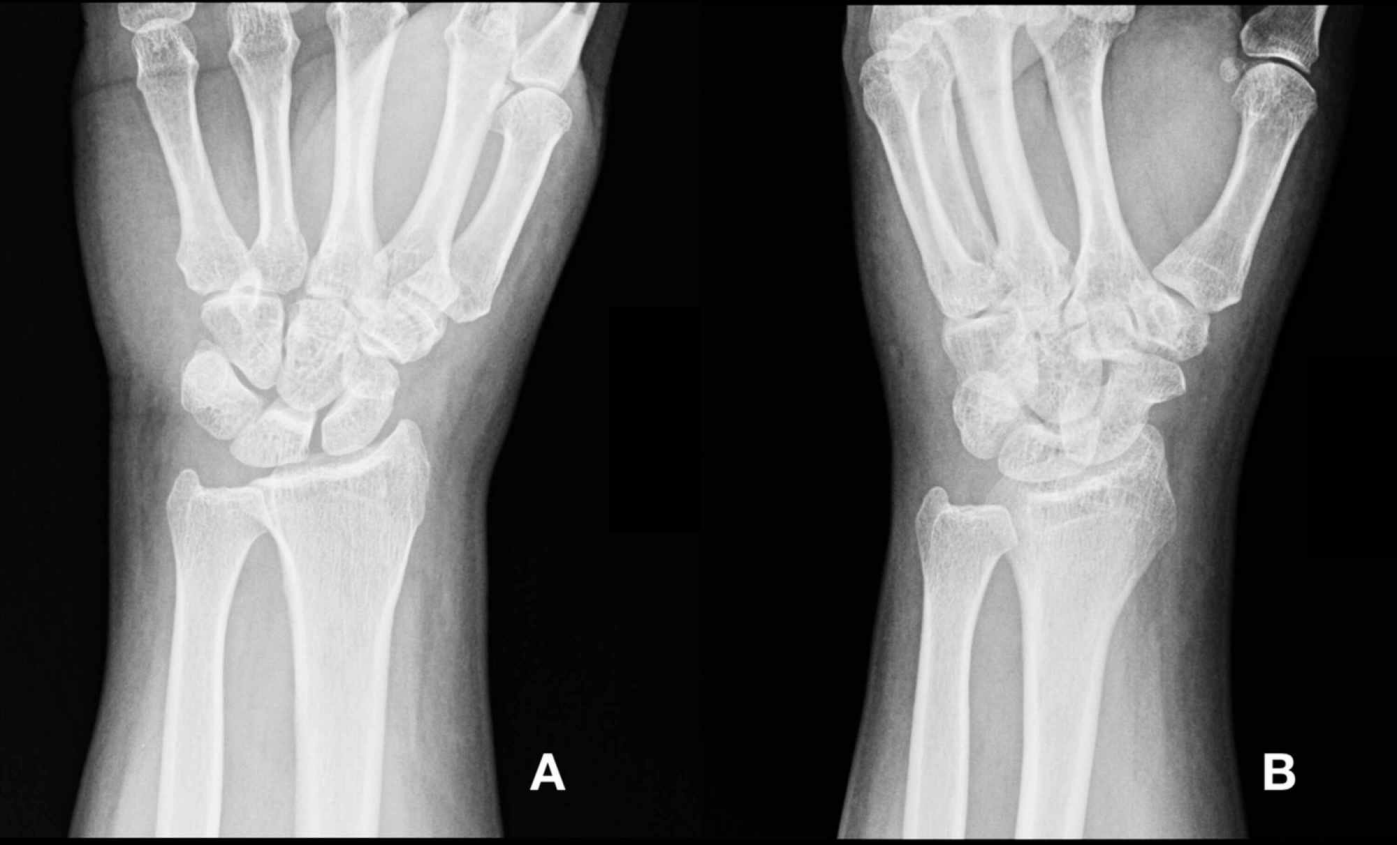
Plain radiograph of the left hand showing mild soft tissue swelling of the wrist. Joint spaces are well maintained. No fracture, dislocation, periosteal reaction, or osteolysis is noted. (A) Anteroposterior view. (B) Oblique view.

Initial workup including an X-ray of the left hand revealed only mild tissue swelling (Figure 1).

Pertinent laboratory values were as follows: white blood cell count (WBC) = 17.2 k/µL, erythrocyte sedimentation rate (ESR) = 88 mm/hr (H), C-reactive protein (CRP) = 23.3 mg/dL (H). Synovial fluid analysis revealed 23,400 cells/mm^3^, 79% polys, 21% mono, and 0 lymphocytes; red blood cell (RBC) = 7,600 cells/mm^3^. No crystals were seen in the synovial fluid, and cultures had no growth.

The patient’s hospital course continued with repeated fever spikes despite being on antibiotics. On day 2 of admission, the pain and swelling in the left wrist completely resolved, and new pain and swelling were noted at the right hip and both ankles. At this time, most investigations including syphilis enzyme immunoassay (EIA), heterophile monospot, antinuclear antibody (ANA), complement levels, rheumatoid factor, anti-cyclic citrullinated peptide (CCP), antineutrophil cytoplasmic antibody (ANCA) levels, gonorrhea/chlamydia urine, pharyngeal and rectal swab, and urine histoplasmosis had returned negative.

On further history taking, the patient reported having a sore throat about a month prior that was partially treated with prescription antibiotics for his grandmother. Taking that history into consideration, an antistreptolysin titer was ordered and returned significantly elevated to 533 units/mL (nl ≤ 200 IU/mL). A transthoracic echo performed to investigate rheumatic heart disease (RHD) did not show valvular pathology.

## Discussion

ARF describes a syndrome of non-suppurative and autoimmune inflammation of joint, muscle, and fibrous tissue that occur as sequela of untreated pharyngitis with GAS [1]. Major manifestations of ARF include carditis, Sydenham’s chorea, subcutaneous nodules, erythema marginatum, and arthritis. Typically, symptoms develop two to three weeks from infection onset and can persist long after the infection has cleared [2]. Although carditis is an important complication of ARF that occurs in 50% to 70% of patients, the migratory polyarthritis seen in ARF is often the first symptomatic sign of disease and occurs in 35% to 66% of patients.

Diagnosing the arthritis of ARF is challenging given its migratory nature and non-specific clinical presentation. As seen in the described patient, his arthritis on presentation closely mimicked septic or gouty arthritis, as it was warm, tender to touch, and monoarticular. Many patients with ARF may present at a time of monoarticular involvement, and thus, clinicians need to have high suspicion for arthralgia migration, and investigate this thoroughly with a detailed history and exam.

Typically, the arthritis of ARF occurs within three weeks of GAS infection. As seen in our patient, arthralgia migration in ARF occurs in quick succession, with each affected joint being inflamed for as short as one to seven days [3]. The arthritis of ARF has a predilection for large joints, and typically affects the lower extremities first. The ankles, knees, elbows, and wrists are all commonly affected. Lastly, ARF arthritis is very responsive to non-steroidal anti-inflammatory drug (NSAID) treatment [2-4]. As seen in our patient, ibuprofen alone was enough to improve swelling, redness, and pain in his affected joints. According to current literature on ARF treatment, if no arthralgia response is seen with NSAIDs after 48 hours, alternate diagnoses should be considered.

In addition to rheumatic fever, the expanded differential for migratory polyarthritis in our patient included the infectious, systemic, and post-infectious arthritides. Infectious causes of joint pain include disseminated gonococcal and non-gonococcal reactive arthritis. While the arthritis of disseminated gonococcal infection can affect large joints, it equally affects small joints causing inflammation, and a characteristic tenosynovitis that differentiates it from other forms of septic arthritis [5]. Septic arthritis is an orthopedic emergency requiring urgent joint washout and antibiotics. On average, admissions for septic arthritis typically last a week [6]. Given the time course of symptom resolution, negative cultures, no recent sexual history, and negative gonorrhea and chlamydia testing, gonococcal and non-gonococcal arthritis was less likely. Systemic causes of arthritis include rheumatoid and systemic lupus erythematosus. Both typically have symmetric joint involvement, morning stiffness, and can appear to have a migratory presentation. However, negative serology for ANA, rheumatoid factor, anti-CCP, and normal C3 and C4 levels made these conditions less likely.

All patients with suspected ARF arthritis should be evaluated for post-streptococcal reactive arthritis (PSRA) as this differentiation carries important consequence. Both ARF and PSRA are types of migratory polyarthritis that occur after untreated pharyngitis with GAS [1,7]. PSRA was first described in 1952 as a clinical entity separate from ARF [7]. PSRA describes the syndrome of polyarthritis that occurs after streptococcal pharyngitis when Jones criteria of ARF are not met. Still, differentiating between the two is clinically challenging. ARF typically occurs in young adolescent patients, while PSRA has a bimodal age distribution occurring at ages 8-14 years and 21-37 years [1]. Arthralgia presentation in ARF typically occurs around three weeks from pharyngitis and has a duration of two to three weeks. In PSRA the arthralgia presents earlier, typically around 10 days, and can last up to two months after pharyngitis [8]. Patients with PSRA typically have associated or isolated polytendonitis, tenosynovitis, or enthesitis. Differing from PSRA, the arthralgia of ARF often responds dramatically to aspirin and NSAIDs. Additionally, acute phase reactants (ESR, CRP) tend to be more dramatically elevated in ARF compared to PSRA.

Unlike PSRA, patients with untreated ARF are at risk of developing RHD and need secondary prophylaxis. The cell wall of Streptococcus bacteria carries a highly antigenic M protein that closely mirrors proteins found on myocardial cells [3]. Through a type II hypersensitivity reaction, antibodies formed against strep bacteria cross-react with myocardial cells, causing inflammation and subsequent carditis [4]. For this reason, the need for long-term prophylaxis against RHD in ARF is strongly supported. To date, the risk of chronic heart disease in PSRA is less clear. A study by Van Bemmel et al, investigating development of valvular heart disease in patients with PRSA showed no increase in risk after a median 8.9-year follow-up [3]. The American Heart Association currently recommends one year of secondary prophylaxis after PSRA diagnosis. If no clinical evidence of carditis is seen during this period, the antibiotics can be stopped [9].

With migratory polyarthritis, fever, and elevated acute phase reactants, our patient fulfilled one major and two minor components of the Jones criteria, and was diagnosed with ARF. PSRA was considered among other differentials. However, considering our patient’s quick response to NSAIDs, and his presentation satisfying the Jones criteria, ARF was deemed more likely. The patient was started on scheduled ibuprofen 800 mg TDS and had resolution of his arthritis over the next two days. He was discharged on prophylactic IM penicillin benzathine.

## Conclusions

Diagnosing the arthritis of ARF is challenging given its non-specific presentation, and onset weeks after pharyngitis. For this reason, clinicians should thoroughly investigate for history of pharyngitis and migratory arthralgia in patients with acute joint pain. This case serves as an opportunity to review the current literature on ARF and highlights both clinical and objective investigations that differentiate ARF from its mimickers. Differentiating the arthritis of ARF from similar presenting arthralgias, specifically PSRA, is important and will guide decision making in regards to secondary prophylaxis against RHD.
